# Viral persistence, reactivation, and mechanisms of long COVID

**DOI:** 10.7554/eLife.86015

**Published:** 2023-05-04

**Authors:** Benjamin Chen, Boris Julg, Sindhu Mohandas, Steven B Bradfute, RECOVER Mechanistic Pathways Task Force, Zaki A Sherif, Christian R Gomez, Thomas J Connors, Timothy J Henrich, W Brian Reeves, K Coombs, C Kim, Pras Jagannathan, Christian Bime, Erin Burke Quinlan, Michael A Portman, Maria Laura Gennaro, Jalees Rehman

**Affiliations:** Department of Biochemistry & Molecular Biology, Howard University College of MedicineWashingtonUnited States; Division of Lung Diseases, National Institutes of Health (NIH), National Heart Lung and Blood Institute (NHLBI)BethesdaUnited States; Department of Pediatrics, Division of Critical Care, Columbia University Vagelos College of Physicians and Surgeons and New York - Presbyterian Morgan Stanley Children's HospitalNew YorkUnited States; Division of Experimental Medicine, University of California, San FranciscoSan FranciscoUnited States; Department of Medicine, Joe R. and Teresa Lozano Long School of Medicine, University of TexasSan AntonioUnited States; NIH RECOVER Research Initiative: Patient representativeBronxUnited States; NIH RECOVER Research Initiative: Patient representativeBronxUnited States; Division of Infectious Diseases and Geographic Medicine, Department of Medicine, Stanford UniversityStanfordUnited States; Division of Pulmonary, Allergy, Critical Care & Sleep Medicine, Department of Medicine, University of Arizona College of MedicineTucsonUnited States; National Center for Complementary and Integrative Health, National Institutes of HealthBethesdaUnited States; Seattle Children’s Hospital, Division of Pediatric Cardiology, Department of Pediatrics, University of WashingtonSeattleUnited States; Public Health Research Institute and Department of Medicine, Rutgers New Jersey Medical SchoolNewarkUnited States; Department of Biochemistry and Molecular Genetics, University of Illinois, College of MedicineChicagoUnited States; 1 Division of Infectious Diseases, Department of Medicine, Icahn School of Medicine at Mount Sinai New York United States; 2 Infectious Diseases Division, Massachusetts General Hospital, Ragon Institute of Mass General, MIT and Harvard Boston United States; 3 Division of Infectious Diseases, Department of Pediatrics, Children’s Hospital Los Angeles, Keck School of Medicine, University of Southern California Los Angeles United States; 4 Center for Global Health, Department of Internal Medicine, University of New Mexico Health Sciences Center Albuquerque United States; DaVita Labs United States; Icahn School of Medicine at Mount Sinai United States

**Keywords:** SARS-CoV-2, long COVID, viral persistence, Reactivation, PASC

## Abstract

The COVID-19 global pandemic caused by the severe acute respiratory syndrome coronavirus 2 (SARS-CoV-2) infection has infected hundreds of millions of individuals. Following COVID-19 infection, a subset can develop a wide range of chronic symptoms affecting diverse organ systems referred to as post-acute sequelae of SARS-CoV-2 infection (PASC), also known as long COVID. A National Institutes of Health-sponsored initiative, RECOVER: Researching COVID to Enhance Recovery, has sought to understand the basis of long COVID in a large cohort. Given the range of symptoms that occur in long COVID, the mechanisms that may underlie these diverse symptoms may also be diverse. In this review, we focus on the emerging literature supporting the role(s) that viral persistence or reactivation of viruses may play in PASC. Persistence of SARS-CoV-2 RNA or antigens is reported in some organs, yet the mechanism by which they do so and how they may be associated with pathogenic immune responses is unclear. Understanding the mechanisms of persistence of RNA, antigen or other reactivated viruses and how they may relate to specific inflammatory responses that drive symptoms of PASC may provide a rationale for treatment.

## Introduction

The Mechanistic Pathways task force within the National Institutes of Health Researching COVID to Enhance Recovery (RECOVER) consortium seeks a mechanistic understanding of post-acute sequelae of severe acute respiratory syndrome coronavirus 2 (SARS-CoV-2) infection (PASC), also known as ‘long COVID,’ to guide treatments. In a majority of individuals infected with SARS-CoV-2, live virus is fully cleared within days to a few weeks after infection and no longer detectable in the respiratory system. However, resolution of viral RNA or antigen from the respiratory epithelia or other tissue sites can be slow, and in some cases viruses may persist in forms that are not well understood. Fundamental questions regarding the relationship between persistence of SARS-CoV-2 and longevity of symptoms of PASC are unresolved. SARS-CoV-2 infection can result in long-term symptoms even if the infection is mild or asymptomatic. There is a wide range of PASC symptoms that can affect different organ systems, including cardiac, respiratory, and neurological processes. Most clinical monitoring of the virus has been conducted in the respiratory tract, although other compartments have been found to harbor SARS-CoV-2. Interestingly, inflammatory responses to SARS-CoV-2 infection can co-exist with re-activation of herpesvirus infections, such as Epstein–Barr virus (EBV). In particular, EBV has been suggested to be associated with chronic fatigue, brain fog, or other symptoms that may resemble PASC symptoms. In fact, infection with other viral or bacterial pathogens has been linked to the development of chronic symptoms in a subset of infected individuals (Bannister, 1988; Keita et al., 2019; Komaroff, 2006; Rebman and Aucott, 2020; Wilson et al., 2018; Zaid et al., 2018). Viral persistence in so-called ‘sanctuary tissues’ has been demonstrated for Ebola virus (Keita et al., 2019; Lo et al., 2017), even after the virus has cleared from blood, persisting for months to years and in some cases inducing chronic symptoms or reactivation of live Ebola virus that results in new outbreaks (Clark et al., 2015; Keita et al., 2021; Wilson et al., 2018). It is still unclear whether SARS-CoV-2 persistence and latent pathogen reactivation plays a role in PASC, and if so, what mechanisms are involved.

**Figure 1.**
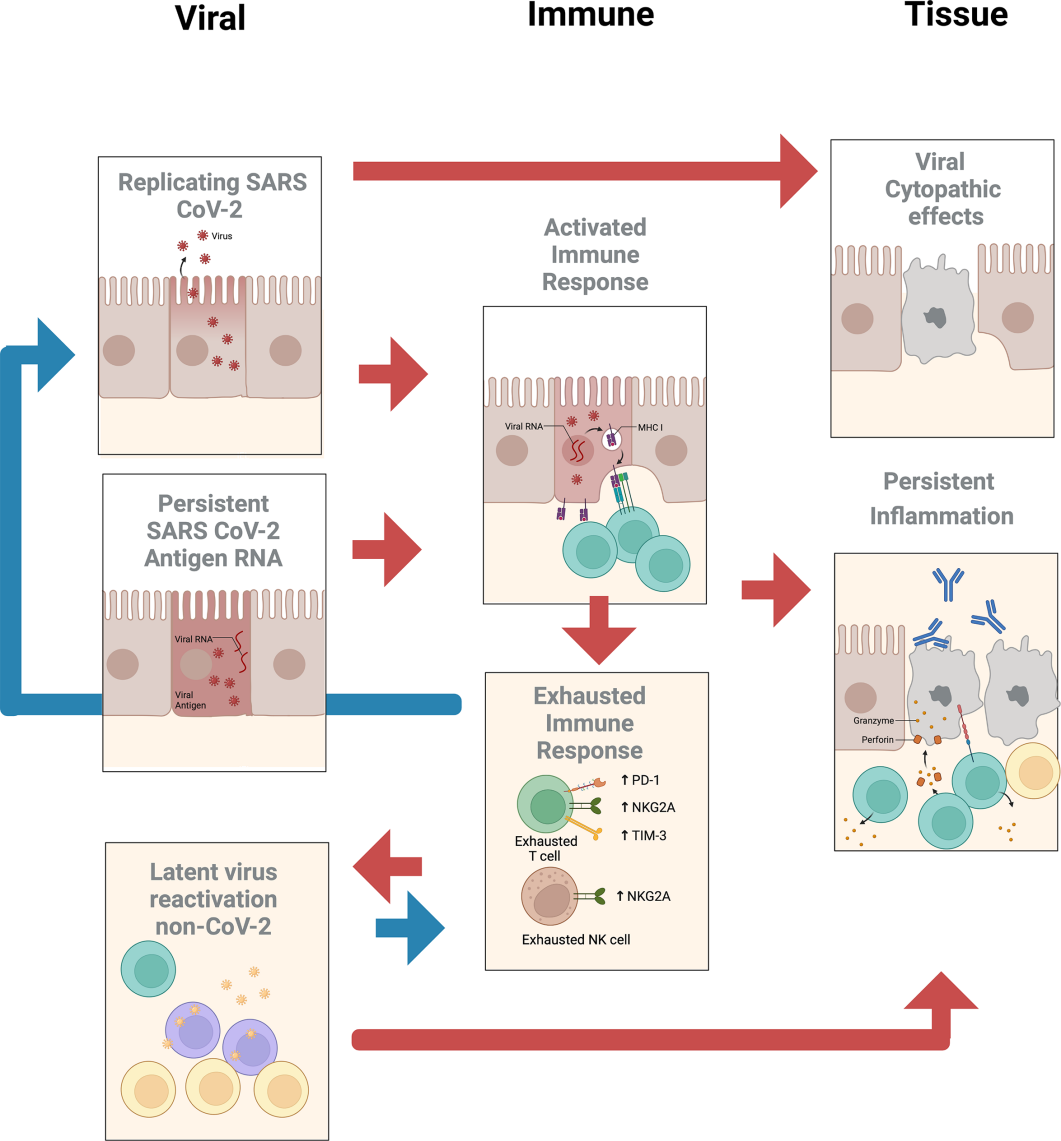
Summary of potential viral, immune, and tissue roles in post-acute sequelae of SARS CoV-2 infection (PASC).

The subcommittee identified two priority areas of research with several subtopics (see Figure 1) that may help determine whether treatment of PASC should employ vaccines or drugs that target SARS-CoV-2 or other reactivated pathogens.

## SARS-CoV-2 viral persistence

### Tissue tropism

SARS-CoV-2 primarily infects individuals through the respiratory epithelium by entering cells that carry the ACE-2 receptor and TMPRSS2 coreceptor in the nasal or upper respiratory epithelium (Clausen et al., 2020; Hoffmann et al., 2020; Melms et al., 2021; Shang et al., 2020; Sungnak et al., 2020; Yan et al., 2020). Diverse cell types within different organ systems express receptors that may make these cells directly vulnerable to infection by SARS-CoV-2, including alveolar macrophages; renal tubular cells; gastrointestinal epithelia in the ileum, colon, and rectum; esophageal keratinocytes; and liver cholangiocytes (Delorey et al., 2021; Gupta et al., 2020; Puelles et al., 2020; Qi et al., 2020; Delorey et al., 2021). Other cells including neurons in the brain are also observed to be infected and may express low levels of ACE-2 or may use alternative coreceptors (Song et al., 2021). Autopsy studies in acute infection show infection of cells in the olfactory mucosal cells including some neurons and endothelial cells within the brain parenchyma (Matschke et al., 2020; Meinhardt et al., 2021). Other single-cell studies of SARS-CoV-2 in the brain reveal no molecular traces of virus in the brain, but they did detect inflammation in choroid plexus cells and find T cells infiltrating the brain (Yang et al., 2021). The existence of diverse viral targets of infection can explain the symptoms of acute infection, though how the impact of acute infection leads to the prolonged symptoms associated with PASC is still uncertain.

### Characteristics of persistent SARS-CoV-2

SARS-CoV-2 RNA can persist for many weeks in the respiratory tract of individuals that have recovered clinically from COVID-19 (Cevik et al., 2021), though the period during which one can isolate infectious virus is much more limited. The persistence of viral RNA in compartments has been primarily measured from the different compartments that shed virus or are easily accessible including the respiratory tract, gastrointestinal tract, and blood. An extensive meta-analysis by Cevik et al. indicates that shedding of virus (as detected by PCR) can continue for prolonged periods in a broad sampling of the population recovering from SARS-CoV-2 infection. The mean duration of shedding is 17 days in the upper respiratory tract, 14.6 days in the lower respiratory tract, 17.2 days in the gastrointestinal tract (fecal), and 16.6 days in blood. The maximum duration of viral RNA shedding was 83 days in the upper respiratory tract, 59 days in the lower respiratory tract, 126 days in stool samples, and 60 d in serum samples. The detection of RNA in the blood during the acute phase of COVID-19 has been correlated with risk of PASC, though in these patients, RNA is generally barely detectable after several months (Su et al., 2022). This may indicate that broad seeding of different organ systems may lead to complications during the recovery. A major question regarding persistence of symptoms is whether the persistence of virus in different compartments may correlate with PASC symptoms. For instance, while there is evidence for infection of olfactory neurons, endothelial cells, and infiltration of immune cells in the CNS, the relationship of persistence of symptoms in the CNS and if they are driven by virus or antigens in the CNS or systemically is still unclear.

Little is known regarding the presence of SARS-CoV-2 viral particles when RNA for SARS-CoV-2 is detectable. Is persisting virus replicating or non-replicating? Most studies that address this issue have been designed to examine the infectiousness of individuals whose fluids test positive for viral RNA. The ability to culture virus from secretions generally is limited to the earlier phases of recovery (Bullard et al., 2020; Liu et al., 2020; Wölfel et al., 2020), except in those with significant immune defects that may enable persistent infection. The ability to sample tissue sites may be different from respiratory, fecal, or blood-derived samples that are relatively simple to collect.

In peripheral blood monocytes, persistence of S1 antigen has been detected by antibody and mass spectrometry in 4 out of 11 severe COVID-19 patients and 1 out of 26 PASC patients, in some cases up to 15 months post infection. In this study, only fragmented RNA could be detected, suggesting that the antigen is not being expressed actively from these cells (Patterson et al., 2021). Viral RNA has been detected in patients with persistent symptoms after COVID-19 in the blood, stool, and urine (Tejerina et al., 2022). The detection of RNAemia and soluble spike antigen in peripheral blood has been associated with PASC in a small group of post-acute COVID patients (Craddock et al., 2023; Craddock et al., 2022). Using ultrasensitive ELISA 60% of individuals with PASC had detectable plasma Spike antigen (Swank et al., 2023). Larger studies are needed to determine whether detection of antigen in these patients is associated with particular symptoms during PASC. In patients with long-lasting olfactory loss, viral RNA could be detected in cells obtained from cytobrush samples but not from routine swabs, and detection of N protein correlated with signs of local inflammation (de Melo et al., 2021). An absence of subgenomic RNA was noted that may be indicative of a lack of active local replication.

Analysis of biopsies from the gastrointestinal tract by immunofluorescence against S and by PCR suggested that antigen persisted in the small bowel in 7 of 14 individuals who were asymptomatic at 4 months after the onset of coronavirus disease (Gaebler et al., 2021). In patients with GI PASC, it has been described that new effector T cell responses may correlate with prolonged symptoms, perhaps indicating that an immune stimulus is still present (Su et al., 2022). In these studies, the authors found persistence of RNA and antigen in 7/14 asymptomatic individuals at 4 months after the onset of COVID-19. Persistence of viral RNA in the gastrointestinal tract has also been found in 32 of 46 patients with inflammatory bowel disease, with viral nucleocapsid detected in 24 of 46. Viral antigens were not detectable in stool and live virus could not be cultured (Zollner et al., 2022). Post-acute sequelae were reported in a majority of patients with antigen persistence but not from those without antigen persistence, leading the authors to conclude that viral antigen persistence forms a basis for post-acute COVID-19. The use of biopsies to assess the low-level persistence of RNA, protein, or active replication-competent virus is not easily performed on some tissues that may be involved in PASC such as the brain, lung, or gastrointestinal tract. Obtaining tissues from symptomatic PASC and asymptomatic patients would be enabled by RECOVER and could lead to a better understanding of the beneficial or pathogenic role(s) of this type of persistent antigen.

The typical half-life of mRNA in the cell is generally thought to be short lived, measured in minutes to hours rather than months or years, and there are not thought to be major exceptions for specific RNAs (Sharova et al., 2009). Thus, the persistence of SARS-CoV-2 RNA is mechanistically a mystery. To date, there is no clear evidence of active viral replication in PASC, though an open question is whether there is persistent viral antigen inducing inflammatory responses within tissues, lungs, brain, and heart. If so, why are these cells that are expressing residual antigen not cleared? Is persistent spike-antigen presence triggering cross-reactive immune responses, similar to other post-infectious syndromes? It should be noted that highly sensitive and specific, reliable, quantifiable, and reproducible detection of viral antigens and/or viral nucleic acids is critical to answer these questions. This is true for SARS-CoV-2 but also for other potentially reactivating viruses (see sections below). Nucleic acid-based detection methods fulfill some of these criteria, but contamination can significantly affect the results. Antibody-based detection of antigens, that is, in tissues, in contrast might be more robust but does not typically achieve the same sensitivity and specificity as nucleic acid detection methods (Cassedy et al., 2021). Spurious results therefore carry the risk for misinterpreting the causal relationship between virus persistence and PASC. Close collaboration between laboratories that study viral persistence and ideally coordinated assay qualification would help reduce and hopefully eliminate these risks. The RECOVER study therefore provides a unique opportunity for such an approach.

### Adaptive/innate immune responses

Repeated antigen exposure is a critical component for the induction of robust and durable adaptive immune responses. In fact, antigen-specific T and B cell populations usually contract once the inducing antigen is cleared. Ongoing evolution of antigen-specific immunity can therefore be evidence for chronic and/or latent infection or persistent antigen presence in the absence of active viral replication, resulting in chronic stimulation of adaptive immune responses. There is a comprehensive body of evidence suggesting that titers of IgM and IgG antibodies against the receptor-binding domain (RBD) of the spike protein of SARS-CoV-2 significantly decrease over time while SARS-CoV-2-specific CD4+ and CD8+ T cell response remain more stable (Bilich et al., 2021; Ibarrondo et al., 2020; Seow et al., 2020; Yamayoshi et al., 2021). In contrast, in a cohort of 87 individuals the frequency of RBD-specific memory B cells appeared to remain stable after SARS-CoV-2 infection and memory B cells displayed ongoing clonal turnover after ~6 months following infection, expressing antibodies with greater somatic hypermutation, indicative of continued evolution of the humoral response (Cho et al., 2021). Furthermore, in a subset of this cohort, persistence of SARS-CoV-2 nucleic acids and immunoreactivity in the small bowel was observed, suggesting that humoral evolution might be linked to antigen persistence. For T-cell responses, in contrast, it has recently been reported that the T-cell receptor (TCR) repertoire diversity decreases quickly after recovering from COVID-19 without changing the global frequency of VDJ gene usage (Luo et al., 2021). SARS-CoV-2-specific CD8+ T-cell responses of significantly increased breadth and magnitude, however, were observed in a small subset of individuals who remained positive for SARS-CoV-2 via RT-PCR nasopharyngeal testing up to 90 d following acute COVID-19, suggesting that such subjects might still harbor replicating virus that drives T-cell activation and evolution (Vibholm et al., 2021). Moreover, cytotoxic CD8+, but also CD4+ T-cell populations, were significantly enriched in individuals with gastrointestinal PASC, suggesting ongoing replenishment and clonal expansion of these cell populations associated with long COVID (Su et al., 2022). Therefore, an unresolved, overarching question is whether viral persistence correlates with evolution of antibody/T-cell responses or inflammatory innate immune responses in PASC.

Severe COVID-19 can result in decreased frequencies and functional exhaustion of antiviral cytotoxic lymphocytes, including cytotoxic T lymphocytes (CTLs) and natural killer (NK) cells, as well as diminished frequencies of plasmacytoid dendritic cells (pDCs) (Diao et al., 2020; Files et al., 2021; Ren et al., 2021; Zheng et al., 2020). In fact, postmortem autopsies of individuals with COVID-19 found a general deficiency of T cells and B cells in lung infiltrates, while innate immune cells were enriched (Duan et al., 2020). In contrast, most individuals following resolution of COVID-19 display a robust memory CD4+ and CD8+ T-cell response toward diverse SARS-CoV-2 viral proteins that are detectable for at least several months after symptom onset (Cohen et al., 2021). Memory lymphocytes, however, can generally be divided into circulating memory cells, found in peripheral blood, and tissue-resident memory cells that reside in the non-lymphoid tissues (Jarjour et al., 2021; Weisberg et al., 2021). Shifts in the lymphocyte distribution leading to potentially reduced frequencies on SARS-CoV-2-specific effector cells in tissues or accumulation of exhausted effector cells in certain anatomic compartments, unable to control or clear the virus, could be considered as a potential mechanism for SARS-CoV-2 persistence in these ‘sanctuary sites.’ In fact, while nucleocapsid-specific antibody and T-cell responses were found to be elevated overall in individuals who developed Neuro-PASC, the severity of cognitive deficits or quality-of-life markers correlated with reduced effector molecule expression in memory T cells, suggesting a potential deficiency of these cells to clear the virus in the CNS compartment (Visvabharathy et al., 2021). Consistently, nucleocapsid-specific IFNγ−/CD107a+ and IFNγ+CD8+T-cell responses decreased more rapidly in individuals with PASC than in convalescent controls, suggesting a dysfunctional immune response (perhaps in response to persistent antigen stimulation) as a contributor to the ongoing clinical syndrome (Peluso et al., 2021). Alternatively, viral persistence in tissues might also serve as a chronic trigger for inflammation along with cellular activation that by itself might result in tissue damage causing PASC-related symptoms. Indeed, elevated frequencies of IFN-γ- and TNF-α-producing SARS-CoV-2-specific T cells in individuals with pulmonary PASC were found to be associated with increased systemic inflammation and decreased lung function, suggesting a potential pathogenic role of SARS-CoV-2-specific T cells, that is, via the production of inflammatory cytokines (Littlefield et al., 2022). It therefore remains to be determined whether a lack of effector cells at immune-privileged anatomical compartments leads to viral persistence or whether viral persistence results in chronic immune activation with all its detrimental consequences, including lymphocyte overstimulation resulting in exhaustion complicated by worsening efficacy of the immune response to control or clear the virus. Comprehensive tissue studies in individuals suffering from PASC versus matched controls will therefore be critical to further investigate these questions.

Innate immune responses in PASC are complex and subject of ongoing in-depth studies. Multiple studies have demonstrated that antibodies generated against cytokines and chemokines correlate with COVID-19 severity and PASC, but a clear consensus on when these responses are protective or pathogenic has been elusive. It appears likely that early type I IFN responses are required for protection against severe acute disease, but delayed responses could be pathogenic (Chiale et al., 2022). Chronic elevated expression of type I IFN and certain pro-inflammatory cytokines are known to cause neurological dysfunction and are found in PASC (Tan et al., 2022). In addition, cellular transcriptional changes are observed in PASC versus non-PASC individuals after resolution of acute disease (Ryan et al., 2022). It is possible that persistence of SARS-CoV-2 RNA and/or protein can lead to chronic activation of PAMPS and innate immune responses, resulting in some of the long-term symptoms found in PASC. Further studies should focus on these mechanisms and possible therapeutic approaches to intervene in the disease course. This topic is more thoroughly discussed in the accompanying review (‘Immune mechanisms underlying COVID-19 pathology and post-acute sequelae of SARS-CoV-2 infection’).

### Long-term shedding and PASC

Many instances of long-term shedding of SARS-CoV-2 have been reported, even after resolution of symptomatic disease. It is possible that chronic production of live virus, viral RNA, or viral protein could lead to onset of PASC, as mentioned above. If this association is found, then monitoring patients post-infection for shedding would be a first step in identifying those at highest risk for long-term disease for potential intervention. Some efforts have been made to determine whether there is a correlation with PASC and viral shedding. One study reported that there was no association of SARS-CoV-2 shedding in saliva and onset of PASC (Peluso et al., 2021). Viral RNA or antigen was found in the gastrointestinal tract of 7 of 14 asymptomatic individuals months after infection, but was not associated with tissue inflammation, suggesting that the presence of viral material alone may not be sufficient for PASC (Gaebler et al., 2021). Conversely, there was an association between tissue persistence of genomic SARS-CoV-2 in olfactory mucosa and loss of smell in four patients, although no infected non-loss-of-smell controls were included in the study (de Melo et al., 2021). A case report of a single individual demonstrated long-term viral RNA shedding of over 100 d in a patient with PASC affecting multiple organ systems (Omololu et al., 2021), although there is a report of chronic shedding by PCR in a patient without long-term symptoms (Chen et al., 2020).

Therefore, several questions remain regarding long-term SARS-CoV-2 shedding and PASC. First and foremost, is there a clear association between the two? One consideration in answering this question lies on the site of sample acquisition for viral shedding. Nasal swabs or saliva are the most accessible samples for monitoring long-term shedding, but it is possible that long-term viral shedding that occurs in the tissue(s) affected by PASC could be more informative. Furthermore, the nature of long-term SARS-CoV-2 shedding needs clarification: does long-term shedding represent only non-infectious genomic RNA or long-lived viral protein, or does it include live virus replication? Understanding the nature and location of long-term SARS-CoV-2 shedding could lead to insights with potential correlative effects with PASC onset.

### Role of non-neutralizing antibody responses in PASC

In some viral infections, antibodies that bind but do not neutralize the virus lead to antibody-dependent enhancement (ADE), a process that increases viral invasion of cells and leads to more severe pathogenesis (reviewed in Arvin et al., 2020). In SARS-CoV-2 infection, neutralizing antibody responses wane rapidly, although binding antibodies persist (Anand et al., 2021). Other antibody functions, such as directing other immune components to lyse-infected cells or engulf viral particles, are not well-explored in PASC.

ADE has been reported with several viral infections including dengue virus (DENV) and respiratory syncytial virus (RSV) (Dejnirattisai et al., 2010; Kim et al., 1969). ADE can be separated into type I ADE where enhanced antibody-mediated virus uptake into Fc gamma receptor (FcγR)-expressing phagocytic cells results in increased viral infection and replication, and into type II ADE, where disproportionate Fc-mediated effector cell activity as well as immune complex formation induce uncontrolled inflammation and immunopathology (type II ADE) (Lee et al., 2020). Both versions of ADE can be observed when non-neutralizing antibodies or antibodies at sub-neutralizing levels bind to viral antigens without efficiently blocking or clearing the infection. Whether ADE plays a significant clinical role in COVID-19 is still undetermined, but the fact that millions have received COVID-19 vaccines and/or endured (repeated) SARS-CoV-2 infection without evidence of more enhanced disease in the setting of preexisting immunity argues against ADE being a significant contributor to SARS-CoV-2 pathogenesis overall. The question, however, has been raised whether ADE could be involved in the development of PASC, specifically in the setting of waning neutralizing antibody titers following acute infection. Indeed, there is ample evidence that neutralizing antibodies against SARS-CoV-2, either following natural infection or vaccination, decline overtime (Cromer et al., 2022; Evans et al., 2022; Levin et al., 2021). Furthermore, a recent prospective cohort study of individuals with COVID-19 found that IgM and IgG3 levels during primary infection and after 6 months were lower among persons with PASC compared to those without (Cervia et al., 2022), while another study did not find any difference in serial antibody levels between those who did and those who did not develop post-COVID-19 symptoms (Pereira et al., 2021). Whether levels of non-neutralizing antibodies that are able to elicit Fc effector functions and potentially ADE are more stable in general in the circulation or whether such antibodies are enriched in specific anatomic compartments remains to be determined. As ADE requires viral antigen to be present, SARS-CoV-2 persistence in tissues together with insufficient neutralizing antibodies levels or elevated levels on non-neutralizing antibodies could theoretically trigger an ongoing inflammatory cascade; however, thus far, clear evidence for that concept is lacking. Therefore, the overall question of whether antibody-dependent enhancement of SARS-CoV-2 occurs in PASC, given the decreasing neutralizing responses after infection, needs to be answered.

Back-boosting of antibodies that recognize seasonal coronaviruses has been recognized early in the pandemic (Ng et al., 2020) with one study reporting ∼20% of 251 tested individuals possessing non-neutralizing antibodies that cross-react with SARS-CoV-2 spike and nucleocapsid proteins prior to SARS-CoV-2 infection (Anderson et al., 2021). The potential effects of such antibodies on COVID-19 severity still remain unclear. While some evidence suggests that prior exposure to seasonal coronaviruses could be protective against severe COVID-19 (Kaplonek et al., 2021; Sagar et al., 2021), other studies have found that antibody responses to seasonal coronaviruses were not associated with protection against SARS-CoV-2 infections or hospitalizations, or even enriched in severe COVID-19 (Aguilar-Bretones et al., 2021; Anderson et al., 2021; Guo et al., 2021) and could potentially inhibit the formation of SARS-CoV-2-neutralizing and non-neutralizing antibodies (Aydillo et al., 2021). Whether preexistent cross-reactive antibodies could play a role in the development of PASC has not been determined.

## Reactivation of latent pathogens

### Identification of reactivated viruses and other pathogens

An intriguing aspect of PASC is the discovery of reactivation of latent viruses after SARS-CoV-2 infection. It has been shown that EBV, a herpesvirus that infects a majority of individuals and is typically in a latent state, can be reactivated after SARS-CoV-2 infection (Chen et al., 2021; Lehner et al., 2020). Some studies have demonstrated a correlation between EBV reactivation and development of PASC (Hao et al., 2021; Su et al., 2022). There has been evidence of reactivation of other herpesviruses, including cytomegalovirus, herpes simplex virus 1, human herpesvirus 6, and human herpesvirus 7, in *acute* SARS-CoV-2 infection (Drago et al., 2021; Lehner et al., 2020; Su et al., 2022; Xu et al., 2020), although the association with these viruses and development of PASC has not been ascertained (Proal and VanElzakker, 2021). Furthermore, some human endogenous retroviruses (HERVs) have been associated with more severe acute SARS-CoV-2 infection (Simula et al., 2022; Temerozo et al., 2022). Therefore, while a few herpesviruses are known to be reactivated in PASC and other viruses have been found to be upregulated in acute disease, identification of the full range of viral species or nonviral pathogens that can be reactivated or triggered has not been characterized. Performing plasma DNA PCR screening or RNA sequencing in samples from people with PASC should answer the question of which latent pathogens are reactivated in PASC versus non-PASC convalescent individuals. Specifically, what spectrum of viruses is reactivated in PASC? Also of interest is whether the timing of latent virus reactivation relative to symptomatic onset of PASC is relevant.

### Effects and mechanisms of latent viral reactivation in PASC

Given the known correlation between EBV reactivation and risk of developing PASC, an important question is the mechanism(s) involved in this process. Understanding of this mechanism is the first step in identifying potential interventions in at-risk individuals. Known mechanisms of EBV reactivation in non-COVID settings include host/viral miRNAs (Chen et al., 2022), other viral genes, histone modifications, reactive oxygen species, the cellular stress response, and cell transcription factors binding to viral promoters (Sausen et al., 2021). Other potential mechanisms include cytokine-mediated reactivation or loss of immune control. It is possible that if non-EBV latent pathogens are reactivated and associated with PASC, different reactivation pathways may be utilized.

### Role of immune responses against reactivated viruses in PASC

Reactivation of latent viruses has been linked to the dysregulation of the host immune response during acute SARS-CoV-2 infection, that is, by disabling the host type I interferon response via autoantibodies (Acharya et al., 2020), resulting in decreased control of these latent pathogens. In fact, severely immunocompromised individuals are in general more prone to the reactivation of EBV. As an example, patients with respiratory failure admitted to the ICU have been shown to have more frequent EBV reactivation, and higher mortality in the setting of reduced CD8+ lymphocytes counts (He et al., 2017). Nevertheless, these viruses were only detectable in blood during the acute phase of COVID-19 and reactivated viruses were so far not explicitly identified in individuals with PASC. Instead, EBV reactivation was determined by serology measuring viral capsid antigen (VCA) IgM and early antigen-diffuse (EA-D) IgG levels that increase as a consequence of viral activity (Gold et al., 2021). It is therefore unclear whether there is a direct pathogenic effect of the latent viruses or whether a short phase of viral reactivation during acute SARS-CoV-2 infection induces additional inflammatory immune responses, contributing to the development of PASC. The finding of activated CMV-specific CD8+ T cells in individuals with gastrointestinal PASC has been attributed to bystander effect rather than a direct virally induced response as no detectable CMV viremia was observed (Su et al., 2022). Evidence for a potential pathogenic role of immune responses against latent pathogens in PASC, however, comes from recent reports pointing to EBV as a possible causative agent in MS (DeLorenze et al., 2006; Levin et al., 2005) and specifically highlighting (EBV-specific) CD8+ T cells as these cells play a key role in antiviral immunity and dominate the CNS immune infiltrate in MS (Salou et al., 2015; van Nierop et al., 2016). Similarly, myocarditis or related cardiac inflammatory issues identified in some individuals with PASC after acute COVID-19 may be driven directly by SARS-CoV-2 but herpesviruses including HHV-6 have been described as the causes for virus-associated inflammatory cardiomyopathy (Tschöpe et al., 2021). Whether virus-specific T-cell responses are contributing to the pathogenesis is insufficiently understood. Future studies will be needed to gain a deeper understanding of the interplay between the host immune response and reactivated latent pathogens and specifically to explore which triggers throw the usually fine-tuned interaction off balance.

### Conclusions

Identifying effective treatments for PASC and its many manifestations will be greatly aided by a better understanding of the role that viral persistence plays in different patients. Given the ability of coronaviruses to infect and reinfect individuals over a lifetime, viral persistence seems likely to play a role in PASC. There is ample evidence for persistence of SARS-CoV-2 viral RNA and proteins in several tissues, including respiratory tract, GI tract, olfactory mucosa, and the central nervous system. The mechanisms of persistence may involve replication or the action of viral replication machinery; however, exactly how viral components persist in individuals is still unclear. If persistence of antigen requires viral polymerase or protease, this provides a strong rationale for testing antivirals in patients. If the persistence is fully independent of replication, then it may be that vaccines that target more diverse genetic targets or other immunomodulators may be beneficial in targeting persistent virus.

The presence of persistent viral antigen and RNA may be the result of incomplete immunity where holes in the immune response permit viral persistence, but there also appears to be evidence that persistent inflammation as a reaction to persistent antigen is causing pathology. A deeper understanding of the adaptive immune responses, including T-cell and B-cell responses, may provide rationales for targeted therapies either to enhance clearance of persistent antigens, or to limit persistent inflammatory responses that are causing damage.

The evidence for reactivation of other latent viruses also appears to be implicated; these provide a rationale for more extended testing for reactivation of EBV and other viruses during PASC or similar post-viral syndromes. Treatment of these viral co-infections may also provide an avenue for therapeutic trials where evidence of persistent viral activation is present.

A major challenge of the treatment of PASC is that it appears to be a highly diverse condition, potentially with several different pathogenetic mechanisms that each will require a distinct treatment approach. Tissue sampling and studies are also particularly important to have a true understanding of the presence and role of viral persistence. Deeper explorations of the mechanisms of viral persistence should aid in the design of clinical trials to match symptoms with trials of drugs that can target specific pathogenic pathways. Studies such as RECOVER that have comprehensive and longitudinal patient data and samples can help answer some of the questions raised above.
